# Dynamic assessment of long-term survival in survivors with stage III non-small cell lung cancer: a novel conditional survival model with a web-based calculator

**DOI:** 10.3389/fmed.2024.1491337

**Published:** 2025-01-07

**Authors:** Xiangdi Meng, Peihe Wang, Jie Liu, Daqing Sun, Zhuojun Ju, Yuanyuan Cai

**Affiliations:** ^1^Department of Radiation Oncology, Weifang People’s Hospital, Weifang, China; ^2^Department of Radiation Oncology, Gunma University Graduate School of Medicine, Maebashi, Japan

**Keywords:** conditional survival, non-small cell lung cancer, nomogram, overall survival, prognostic factor

## Abstract

**Background:**

Conditional survival (CS) analysis can estimate further survival probabilities based on the time already survived, providing dynamic updates for prognostic information. This study aimed to develop a CS-nomogram to promote individualized disease management for stage III non-small cell lung cancer (NSCLC).

**Methods:**

This study included patients diagnosed with stage III NSCLC in the Surveillance, Epidemiology, and End Results database from 2010 to 2017 (*N* = 3,512). The CS was calculated as CS(y|x) = OS(y + x)/OS(x), where OS(y + x) and OS(x) were the overall survival (OS) in the year (y + x) and year x, respectively, calculated by the Kaplan–Meier method. We used the least absolute shrinkage and selection operator (LASSO) regression to identify predictors and developed the CS-nomogram based on these predictors and the CS formula.

**Results:**

The CS analysis provided real-time updates on survival, with 5-year OS improving dynamically from 14.4 to 29.9%, 47.9, 66.0, and 80.8% (after 1–4 years of survival). Six independent predictors (age, tumor size, N status, surgery, radiotherapy and chemotherapy) were identified for the development of the CS-nomogram and its web version (https://dynapp.shinyapps.io/NSCLC/). The model performed with an excellent concordance index (C-index) of 0.71 (95% CI: 0.70–0.72), and a median time-dependent AUC of 0.71–0.73 from 200 iterations 5-fold cross-validation.

**Conclusion:**

The study demonstrated the improvement in real-time OS over time in stage III NSCLC survivors and developed the novel CS-nomogram to provide patients with updated survival data. It provided novel insights into clinical decisions in follow-up and treatment for survivors, offering a convenient tool for optimize resource allocation.

## 1 Introduction

Non-small cell lung cancer (NSCLC) accounts for approximately 85–90% of all lung cancers (1–6), with 20–30% of cases diagnosed at stage III, a group characterized by substantial heterogeneity (3–7). This heterogeneity poses significant challenges for both treatment and follow-up. Although various treatment strategies, such as surgery, radiotherapy, targeted and immunotherapy, have been used to manage the disease, the prognosis for stage III patients remains unfavorable and individual survival rates vary widely (4, 7–9). Previous studies have confirmed that the real-time prognosis of cancer survivors improved dynamically over time, indicating that initial prognostic assessments at diagnosis may underestimate a patient’s current prognosis (10–12). Unfortunately, the lack of personalized assessment tools has prevented survivors from receiving an up-to-date, personalized prognosis. In addition, traditional survival estimates provide only static data tied to the initial diagnosis, underscoring the need for a dynamic monitoring system that can continuously update individualized survival information throughout follow-up.

Conditional survival (CS) analysis predicts future survival using the time survived (13). Compared with traditional survival analysis, CS analysis provided a relatively accurate estimate of the change in patient prognosis over time and allowed real-time updating of survival data (13–15). CS analysis has been widely used in various cancers to optimize clinical decision-making and reduce psychological distress in survivors (16–19). Additionally, several clinicopathological factors, such as age, tumor stage, and treatment strategy, have been demonstrated to impact the individualized prognosis of NSCLC patients (20). The CS nomogram is a tool based on CS analysis and statistical modeling methods that integrated individualized information and considered survival time, allowing real-time updating of survival data for patients at different follow-up time points (15). However, these methods have never been applied to stage III NSCLC.

This study aimed to elucidate changes in survival over time in patients with stage III NSCLC and to develop an easy-to-use CS nomogram and host it on a website to provide individualized, real-time prognostic information to inform patients of their latest survival data and guide optimal clinical decision making.

## 2 Materials and methods

### 2.1 Patients and variables selection

After obtaining access to the Surveillance, Epidemiology, and End Results (SEER) database, we collected NSCLC patients aged 18 years or older between 2010 and 2017 according to the International Classification of Diseases for Oncology (ICD-O-3) (site code C34 “Lung and bronchus” and morphology code 8046/3 “non-small cell carcinoma”). In addition, we excluded the following patients: (1) not stage III (American Joint Committee on Cancer 7th edition, AJCC 7th); (2) not confirmed by histopathology; (3) not the first primary tumor; (4) survival time less than 1 month; (5) necessary variables (age, race, marriage, gender, staging, surgery) were unknown. For the continuous variable (age), grouping was performed using a restricted cubic spline (RCS) with a cut-off point of 70 years before analysis. Tumor size was divided into groups for every 10 mm, and 70 mm or more was grouped as a separate group. Given the difficulty in determining the number of specific positive lymph node metastases in unoperated patients, lymph node metastases were grouped according to the AJCC 7th N stage. The primary clinical endpoint was overall survival (OS), defined as the time from patient diagnosis to death.

### 2.2 Statistical analysis

Means and standard deviations (SDs) were reported for continuous variables that followed a normal distribution. Otherwise, medians and interquartile ranges (IQRs) were reported. In addition, categorical variables were presented as numbers and percentages of cases. The Kaplan–Meier method was used to estimate overall survival (OS) in patients with stage III NSCLC.

The CS was calculated using the formula CS(y|x) = OS(y + x)/OS(x), where x and y represented the survived time after diagnosis and the expected further survival time, respectively (13). For example, to calculate the real-time survival rate for patients who have survived 2 years after diagnosis for another 3 years would be CS(3|2) = OS(3 + 2)/OS (2), which was 5-year OS divided by 3-year OS. In addition, the study used annual hazard rates to show the annual risk of death after the patient’s diagnosis.

We used the least absolute shrinkage and selection operator (LASSO) regression to screen predictors to improve the underfitting or overfitting of the model and multivariate Cox regression to show the effect of predictors on survival. A nomogram was developed using the screened predictors to estimate individualized survival, and real-time OS was further accurately calculated based on the CS formula to develop a CS-nomogram. After entering the patient’s individualized parameters, the model quantified the predictors as risk scores and calculated the total risk score, which corresponded to the individualized OS and informed the patient of the real-time OS after several years of survival. For ease of use, we deployed its web version, which provided survival predictions accurate to the month. Finally, we evaluated the performance of the CS-nomogram by reporting concordance index (C-index), time-dependent receiver operating characteristic (ROC) curves, calibration plots, decision curve analysis (DCA) curves and 200 times of 5-fold cross-internal validation.

## 3 Results

**Figure 1:**
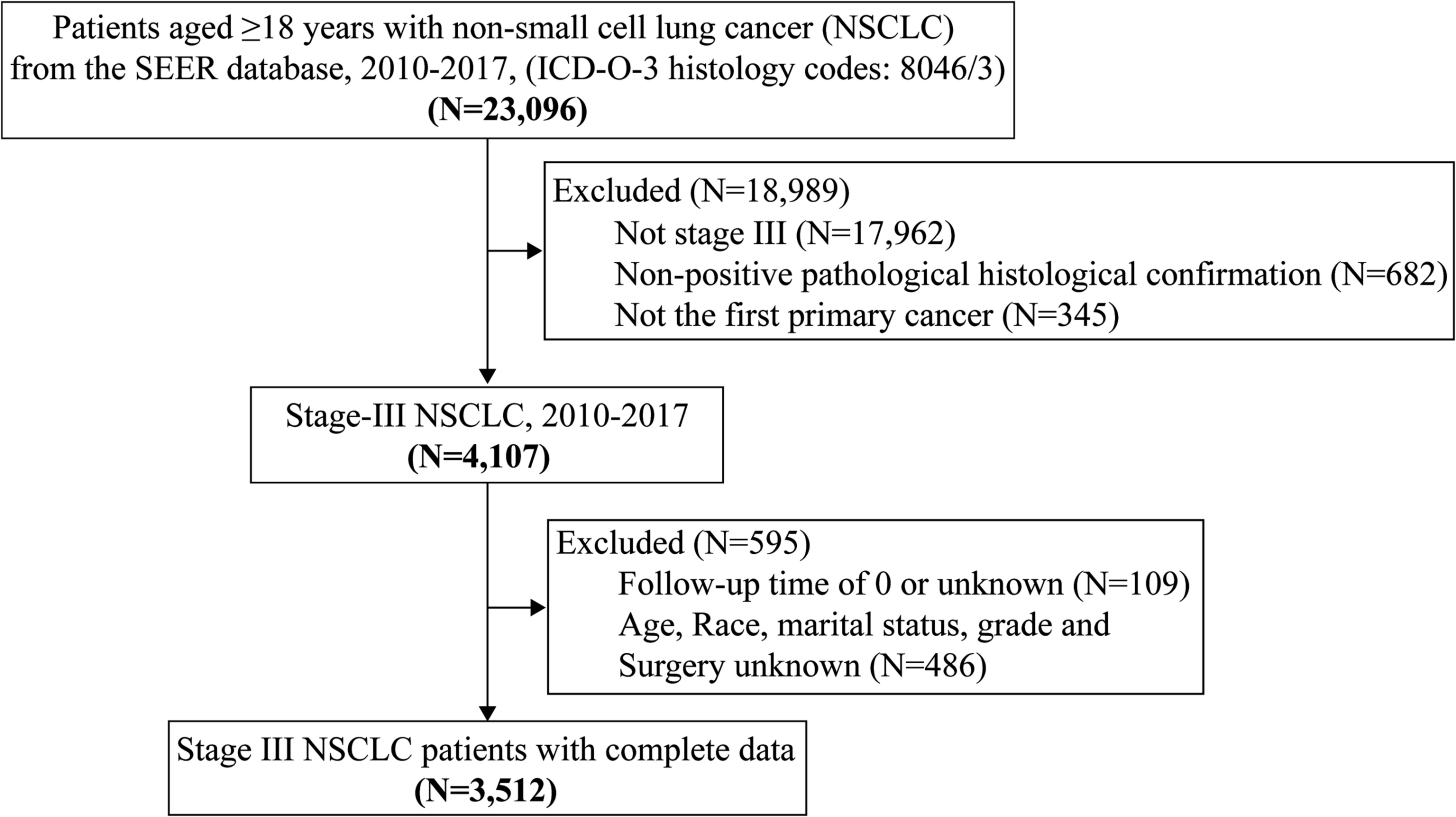
Flow chart for screening patients with Stage III non-small cell lung cancer.

A total of 3,512 patients with locally advanced (stage III) NSCLC were included in the study (Figure 1), with a median age of 69 years (SD = 10.9 years). The majority of patients were of white with 79.9% (2,806/3,512), followed by black with 13.9%. In the whole cohort, 45.4 and 54.6% of patients were classified as stage IIIA and IIIB, respectively. The majority of patients underwent surgery (93%, 3,266/3,512). In addition, 63.0% (2,214/3,512) and 64.5% (2,265/3,512) of patients received radiotherapy and chemotherapy, respectively (Table 1).

**Table 1:** Clinicopathological characteristics of stage III non-small cell lung cancer patients (*N* = 3,512).

Characteristics	Whole cohort
	*N* = 3,512 (%)
Age at diagnosis (mean ± SD)	69.0 ± 10.9
≤70	1,899 (54.1)
>70	1,613 (45.9)
**Race**	
White	2,805 (79.9)
Black	489 (13.9)
Other	218 (6.2)
**Marital status**	
Unmarried	1,696 (48.3)
Married	1,816 (51.7)
**Gender**	
Male	1,968 (56.0)
Female	1,544 (44.0)
**Tumor size (mm)**	
≤10	120 (3.4)
11–20	443 (12.6)
21–30	530 (15.1)
31–40	518 (14.7)
41–50	486 (13.8)
51–60	387 (11.0)
61–70	349 (9.9)
>71	679 (19.3)
**N status (AJCC 7th)**	
N0	509 (14.5)
N1	195 (5.6)
N2	2,144 (61.0)
N3	664 (18.9)
**TNM stage (AJCC 7th)**	
IIIA	1,593 (45.4)
IIIB	1,919 (54.6)
**Surgery**	
No	3,266 (93.0)
Yes	246 (7.0)
**Radiotherapy**	
No	1,298 (37.0)
Yes	2,214 (63.0)
**Chemotherapy**	
No	1,247 (35.5)
Yes	2,265 (64.5)
Follow-up time [median (IQR)]	12 [5, 29]
**Status**	
Alive	463 (13.2)
Dead	3,049 (86.8)

SD, standard deviation; IQR, interquartile range; TNM, tumor-node-metastasis; AJCC, the American Joint Committee on Cancer.

**Figure 2:**
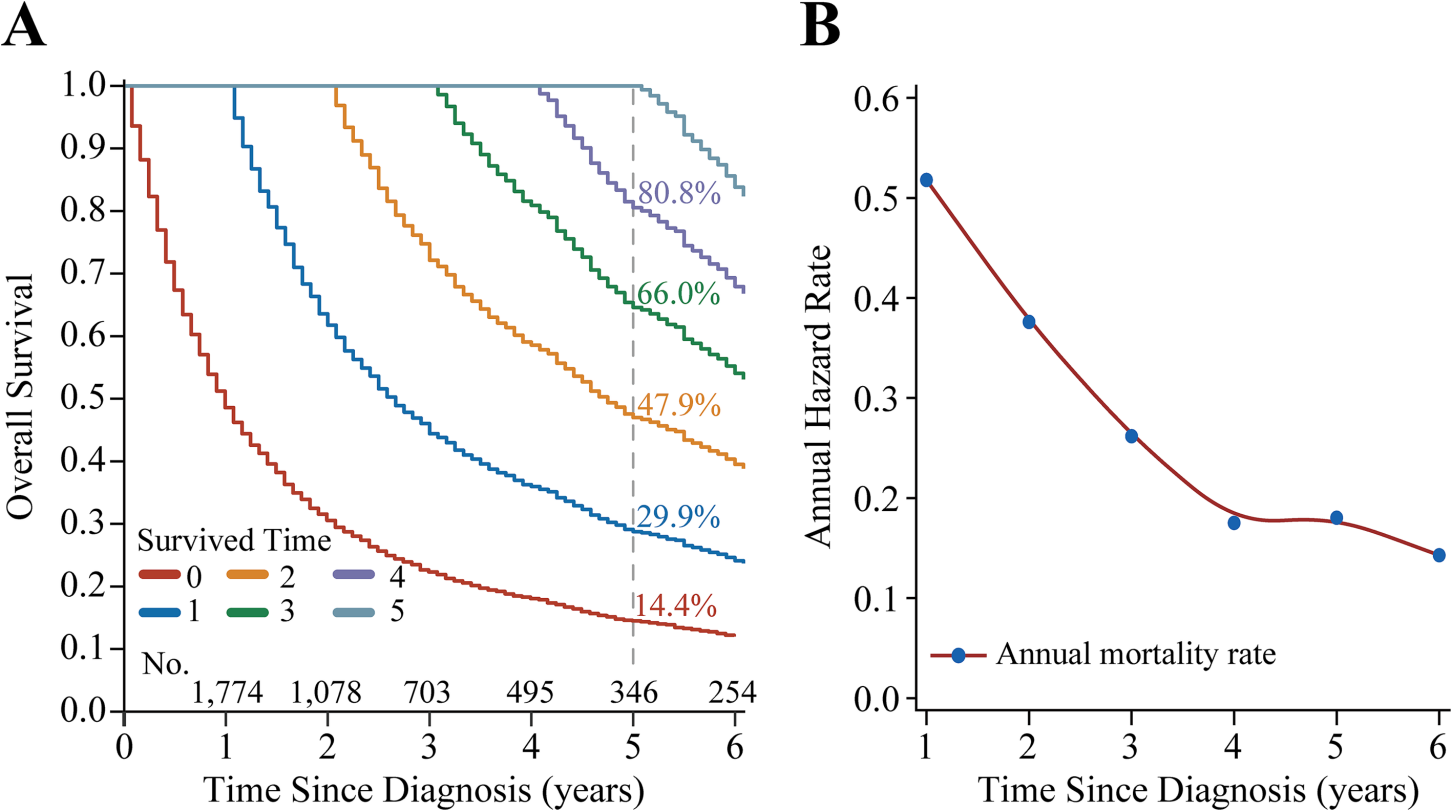
Survival analysis of patients with Stage III non-small cell lung cancer. **(A)** Kaplan–Meier curves estimating real-time survival rates after surviving for 0–5 years. **(B)** Annual hazard rate curve.

The median follow-up of the study was 12 months (IQR: 5 months, 29 months), with 1-, 3-, and 5-year OS of 48.1% [95% confidence interval (CI): 46.5–49.8%], 21.8% (95% CI: 20.5–23.2%), and 14.4% (95% CI: 13.2–15.7%), respectively (Figure 2A). CS analysis showed that the real-time survival estimates of patients after diagnosis gradually improved over time, with 5-year OS increasing from an initial 14.4 to 29.9%, 47.9, 66.0, and 80.8% each year (Figure 2B). In addition, the annual hazard rate curve showed that the all-cause mortality rate for patients with stage III NSCLC decreased each year and remained relatively stable after the fourth year (Figure 2B).

**Figure 3:**
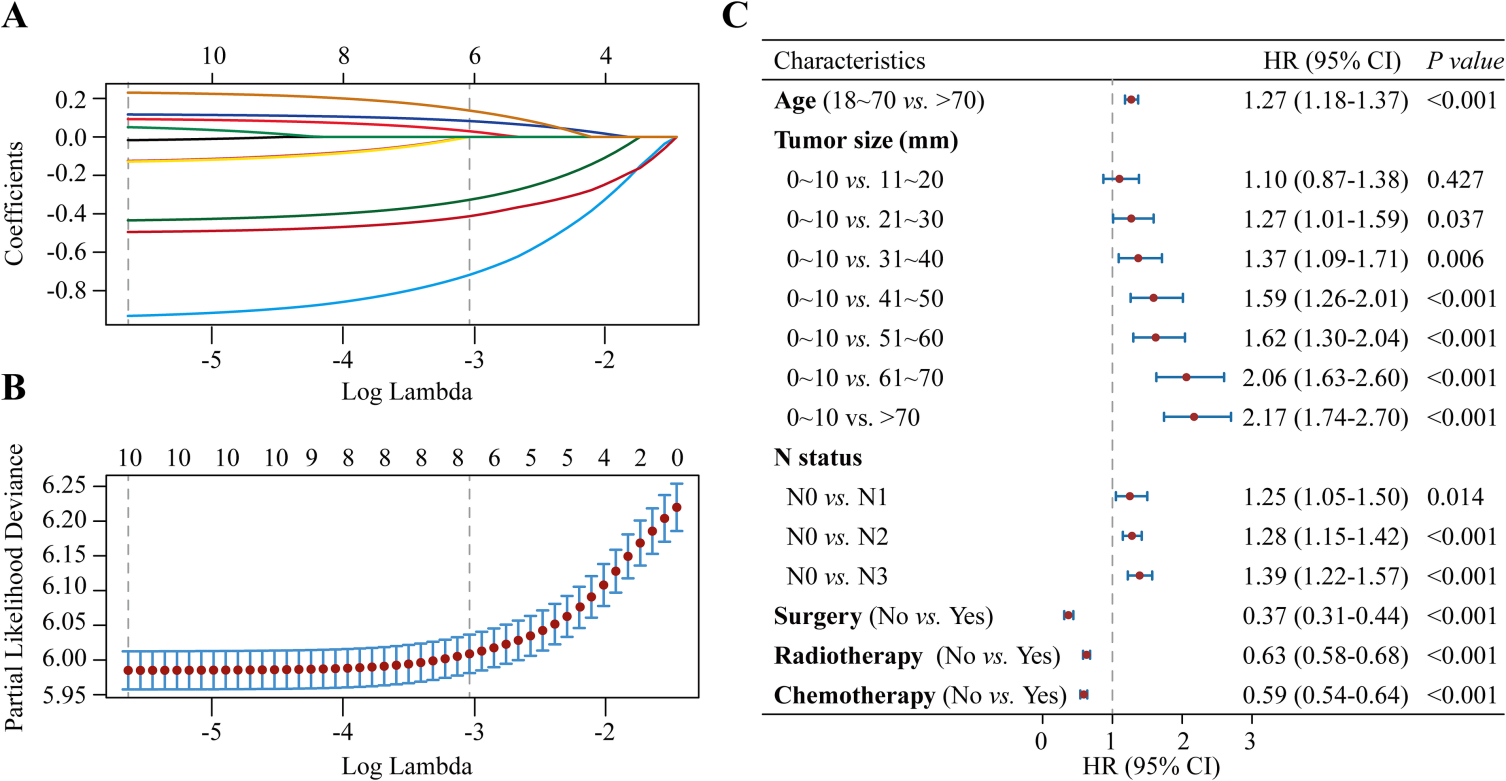
Predictor screening. **(A)** The least absolute shrinkage and selection operator (LASSO) regression with **(B)** 5-fold cross-validation, and **(C)** Multivariate Cox regression forest plot of predictors.

**Figure 4:**
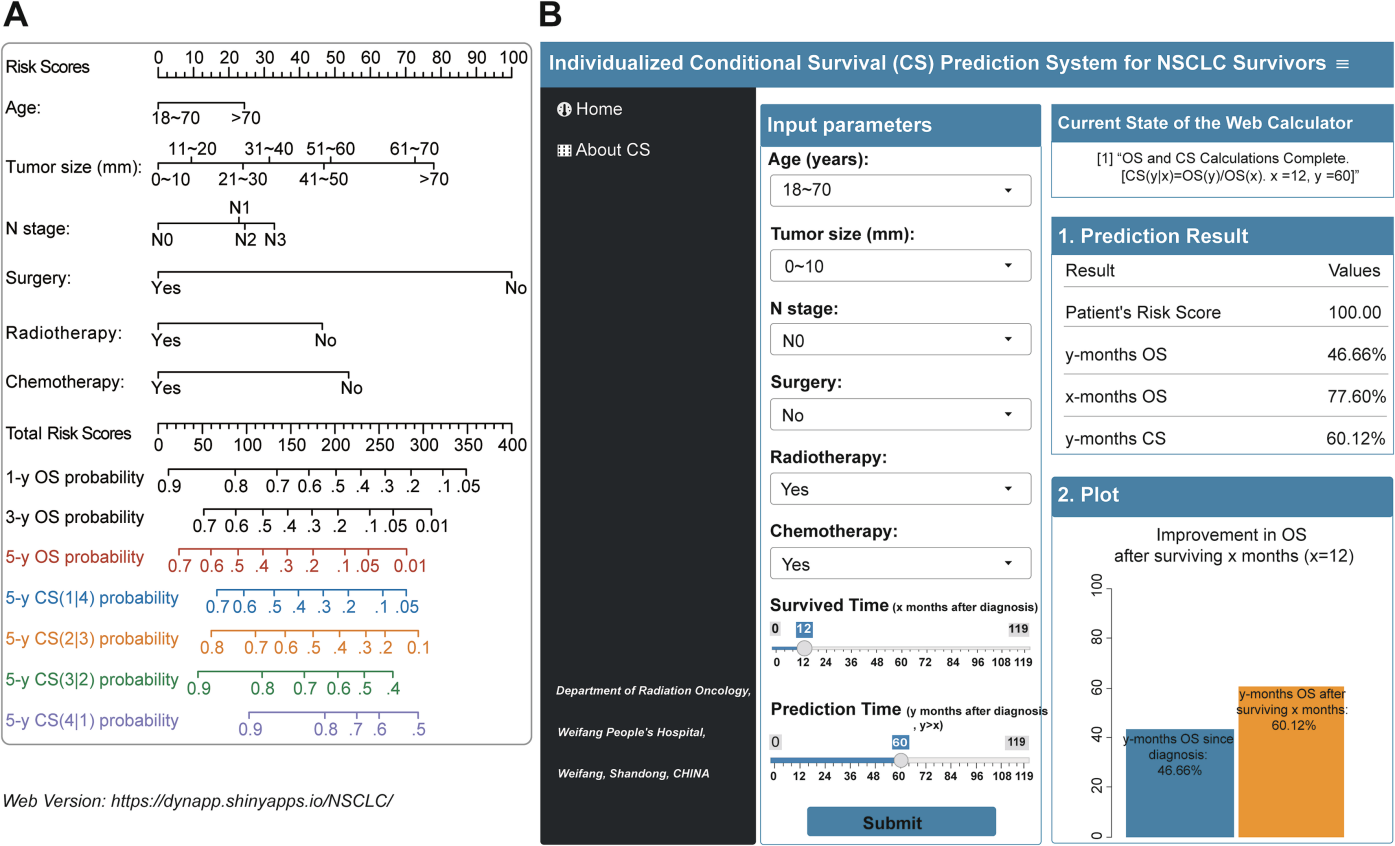
**(A)** Conditional survival nomogram (CS-nomogram) and **(B)** its web version for survivors with Stage III non-small cell lung cancer (https://dynapp.shinyapps.io/NSCLC/). OS, overall survival; CS, conditional survival.

**Figure 5:**
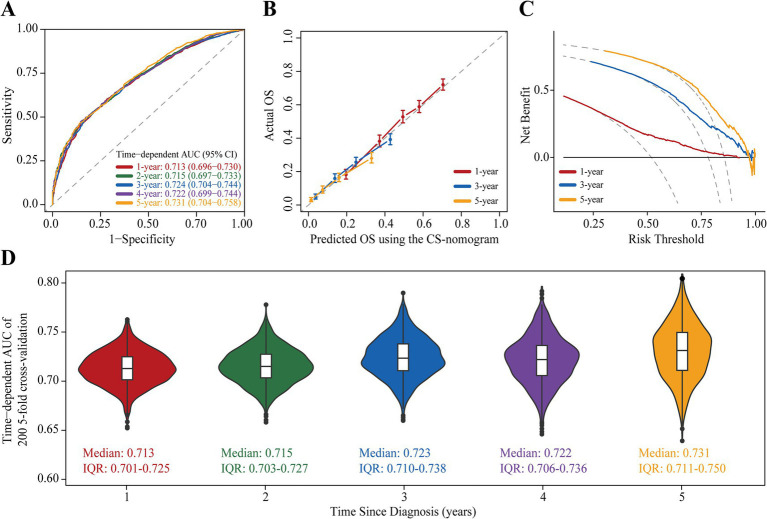
Assessment and Validation of the conditional survival nomogram (CS-nomogram). **(A)** Time-dependent area under curve (AUC); **(B)** Calibration plot and **(C)** decision curve analysis (DCA) curve for assessing the accuracy, discrimination and clinical usefulness of the conditional survival nomogram (CS-nomogram). **(D)** 200 times 5-fold cross-validation for estimating model time-dependent AUCs from 1 to 5 years. IQR, interquartile range.

The study examined the predictors using LASSO regression and found that the model constructed with age, tumor size, N status, surgery, radiotherapy and chemotherapy had a minor error (Figures 3A,B). Meanwhile, multivariate Cox regression showed a significant effect of these six predictors on OS in patients with stage III NSCLC (Figure 3C). Based on the screened predictors, we constructed a nomogram to estimate individualized 1- to 5-year OS and developed the CS-nomogram capable of predicting 5-year CS in real-time (Figure 4A). Furthermore, we deployed a web version of this model (Figure 4B, https://dynapp.shinyapps.io/NSCLC/), enabling easy real-time estimation of patient survival by entering individualized parameters and the time already survived. Notably, the model exhibited good discrimination, as evidenced by a C-index of 0.71 (95% CI: 0.70–0.72), and demonstrated stability over 5 years, with a time-dependent median area under the curve (AUC) of 0.722 across 1–5 years (Figure 5A). The calibration plots demonstrated that the model achieved high accuracy, with curves closely resembling the ideal 45° reference curve (Figure 5B). Additionally, the DCA curves highlighted the clinical utility of the CS-nomogram, showing that utilizing the model to guide clinical interventions outperformed the treat-all or treat-none strategies (Figure 5C). Lastly, the model’s performance exhibited high stability through 200 iterations of 5-fold cross-validation, yielding median values of 0.713 (IQR: 0.701, 0.725), 0.715 (IQR: 0.703, 0.727), 0.723 (0.710, 0.738), 0.722 (0.706, 0.736), and 0.731 (IQR: 0.711, 0.750) for the time-dependent AUC (1–5 years after diagnosis) (Figure 5D).

## 4 Discussion

Stage III NSCLC is notably heterogeneous, and the lack of reliable individualized prognostic estimates poses a significant challenge for disease management. In this study, we applied a novel CS analysis method specifically to stage III NSCLC. Our observations revealed that the OS of patients gradually improved over time. Importantly, the study culminated in the successful development of a CS-nomogram, which has been deployed on a dedicated website to provide personalized, real-time updates on survival data.

NSCLC accounts for 80–85% of all lung cancer cases, with approximately one-third being initially diagnosed at locally advanced stages (6). Stage III NSCLC encompasses a spectrum of different clinical conditions, exhibits significant heterogeneity, and requires a multidisciplinary treatment approach with various therapies (3, 6, 7). Additionally, considerable variability in patient survival rates was observed (9). Our study found the median survival of patients to be only 12 months (95% CI: 12–13 months), corroborating the findings of Flores et al. (8). Advanced age, smoking, and late staging emerged as unfavorable prognostic factors for patients (1, 20–22). To establish an appropriate age stratification cut-off, we conducted an RCS analysis with the objective of achieving a significant difference in OS between the two patient groups, thus enhancing the model’s utility. It was important to note that the SEER database’s incomplete description of T status compounded the challenges of staging conversion between AJCC versions 7 and 8. Consequently, rather than incorporating T status directly into our model, we utilized tumor size as a surrogate marker. Although this method may sacrifice some details, such as the extent of invasion and depth of infiltration, the choice of tumor size as a variable offers greater accessibility, thereby improving our model’s generalizability. To enhance survival outcomes in locally advanced NSCLC, previous research has suggested various treatments, including surgical intervention, concurrent radiotherapy, adjuvant radiotherapy, or chemotherapy for patients with stage III disease. In our model, the absence of surgery corresponded to a quantitative risk score of 100, while the inclusion of radiotherapy and chemotherapy lowered the risk scores by 46.4 and 53.9, respectively. This implied that in cases where surgery was not an option, a combination of radiotherapy and chemotherapy could offer a comparable survival advantage. The heterogeneity of clinicopathologic characteristics and treatments in current cancer research presents a substantial challenge in the implementation of individualized survival estimates (23, 24). Crucially, the lack of a reliable tool for personalized survival estimation had previously placed a significant psychological burden on survivors, leaving them uncertain about their life expectancy. Furthermore, the inability to stratify survivors accurately based on risk led to the need for indiscriminate follow-up of all patients, thereby adding to the strain on healthcare systems. The CS-nomogram developed in this study emerges as a vital tool to alleviate these concerns.

Contrasting with traditional survival estimation methods, CS analysis considers not only the baseline characteristics of patients at diagnosis but also the duration already survived (14). This model offered a more dynamic and individualized approach to survival estimation, enabling physicians to more accurately assess patient prognosis and make informed clinical decisions (25). In our study, we noted a consistent and progressive improvement in real-time survival among patients with stage III NSCLC. Similar trends have been observed in breast (26), esophageal (17, 27), colorectal (14) and lymphoma cancers (28, 29), underlining its significance as a crucial indicator for follow-up. Previous research suggested that this improvement was attributable to a natural selection effect: the occurrence of death among high-risk stage III NSCLC patients led to an increase in the average survival of low-risk individuals, thereby contributing to the ongoing enhancement in real-time survival rates (14).

The CS-nomogram presents the significant advantage of providing real-time updates on individual survival data for survivors during follow-up, marking a substantial improvement over previous method (29). Traditional nomograms are based on the patient’s condition at the time of initial diagnosis, and their 5-year OS does not change with the duration of survival after diagnosis, making them a static prediction tool. In current clinical consultations, patients are increasingly interested in knowing their prognosis after surviving for a certain period of time. This is a common question during routine follow-up. The introduction of the CS model has greatly simplified this issue. For instance, inputting a patient’s clinical parameters and the duration already survived (e.g., 2 years) into the model yields a risk score of 121.17 points, initially corresponding to a 5-year OS of 36.85% at diagnosis. Notably, since the patient has survived for 2 years, the CS model can update the 5-year OS to 66.92% [5-year CS (3|2)]. This updated information significantly boosts patients’ confidence in combating the disease and alleviates their anxiety. Moreover, the clinicopathologic features utilized in the monitoring system have been demonstrated in prior studies to be pertinent in stage III NSCLC, while also being convenient and user-friendly. For clinicians, the CS-nomogram aids in the individualized assessment of patient survival and allows for more targeted follow-up strategies for high-risk patients or those who are potential clinical trial candidates. This undoubtedly enhances patient management and the efficient use of medical resources.

The current study has many limitations to report. First, retrospective bias was inevitable; second, some essential variables, such as smoking history and physical status, were missing from the SEER database, which may have limited some of our analyzes. Third, patient survival will continue to improve with the advent of new therapies, such as targeted agents and immunotherapies, and patient survival may be underestimated. Fourth, although the model was internally evaluated and validated to demonstrate superior performance, external validation is also necessary. Therefore, in future research, we will introduce more useful variables to the existing methods to ensure the performance and generalizability of the model.

## 5 Conclusion

In this study, we demonstrated that real-time OS in stage III NSCLC survivors improved over time. We developed a CS-nomogram and its web-based version to inform patients of their updated survival data, which is expected to bring new guidance to disease management.

## Data Availability

The raw data supporting the conclusions of this article will be made available by the authors, without undue reservation.
